# THE RELATIONSHIP BETWEEN CHILDHOOD ADVERSITY AND MENTAL HEALTH IN OLDER ADULTS

**DOI:** 10.1093/geroni/igac059.2698

**Published:** 2022-12-20

**Authors:** James Lian, Kaarin Anstey, Kim Kiely

**Affiliations:** University of New South Wales, Sydney, New South Wales, Australia; University of New South Wales, Sydney, New South Wales, Australia; University of New South Wales, Sydney, New South Wales, Australia

## Abstract

**Background:**

There is a need to understand how early adversity is linked with mental health in older adults. The aim of this study was to 1) explore the optimal way to operationalise a scale of adverse childhood experiences (ACEs) and 2) examine the association between ACEs with depression and anxiety in older adulthood.

**Methods:**

Data were from Wave 1 of the Personality and Total Health (PATH) Through Life Project (N = 7485, 51% women). Older adults aged 60–65 reported their childhood experiences of domestic adversity on a 17-item scale (e.g., physical abuse, neglect, poverty). Depression and anxiety were assessed using four validated screening instruments (GDS, GAS, MCS-12, PHQ-9). Three approaches to scoring the ACE scale were compared: i) cumulative risk, ii) factor analysis, and iii) latent class analysis (LCA). Confirmatory factor analysis (CFA) of the dimensional model of adversity and psychopathology was examined. Linear regression models estimated associations between ACEs and mental health, adjusting for age, race, education, and gender.

**Results:**

Childhood adversity was associated with late life depression and anxiety using the cumulative risk approach. CFA examined latent factors of threat and deprivation in our dataset, which were highly correlated, leading to problems with multicollinearity when estimating associations. Finally, LCA revealed six classes of ACEs: high adversity, low adversity, low affection, authoritarian upbringing, high parental dysfunction, and moderate parental dysfunction. High adversity and high parental dysfunction were associated with higher levels of depression and anxiety symptoms compared to other latent classes.

